# Supplementary material for: D-Aspartate Depletion Perturbs Steroidogenesis and Spermatogenesis in Mice
Source: Biomolecules. 2023 Mar 30;13(4):621. doi: 10.3390/biom13040621 (PMC10136051; doi:10.3390/biom13040621)
Supplement: Supplementary file 1 [file biomolecules-13-00621-s001.zip › biomolecules-2266297-supplementary.pdf]

Table S1. Antibody for western blot and/or immunofluorescence analysis.

Antibody	WB Dilution	IF Dilution	Sources
anti-17β-HSD	1:1000	1:100	sc-32872; Santa Cruz Biotechnology
anti-PCNA	1:1000	1:100	#98825; Sigma-Aldrich
anti-SYCP3	1:250	-	sc-74569; Santa Cruz Biotechnology
anti-DAAM1	1:1000	1:100	#E-AB-21207; Elabscience Biotechnology
anti-PREP	1:3000	1:100	#ab58988; Abcam
anti-GnRH	1:2000	1:100	sc-32292; Santa Cruz Biotechnology
Anti-Cyt C	1:1000	-	#4272; Cell Signaling
anti-β-actin	1:2000	1:100	#E-AB-20031; Elabscience Biotechnology
α-Tubulin	-	1:100	#E-AB-20036; Elabscience Biotechnology
Goat anti-rabbit HRP	1:3000	-	#AP307P; Sigma-Aldrich
Goat anti-mouse HRP	1:2000	-	#AP130P; Sigma-Aldrich
Goat anti-rabbit Alexa Fluor 488	-	1:500	#A32731; Thermo Fisher Scientific
Goat anti-mouse Alexa Fluor 647	-	1:500	#A21236; Thermo Fisher Scientific
PNA lectin	-	1:50	#L32456; Thermo Fisher Scientific

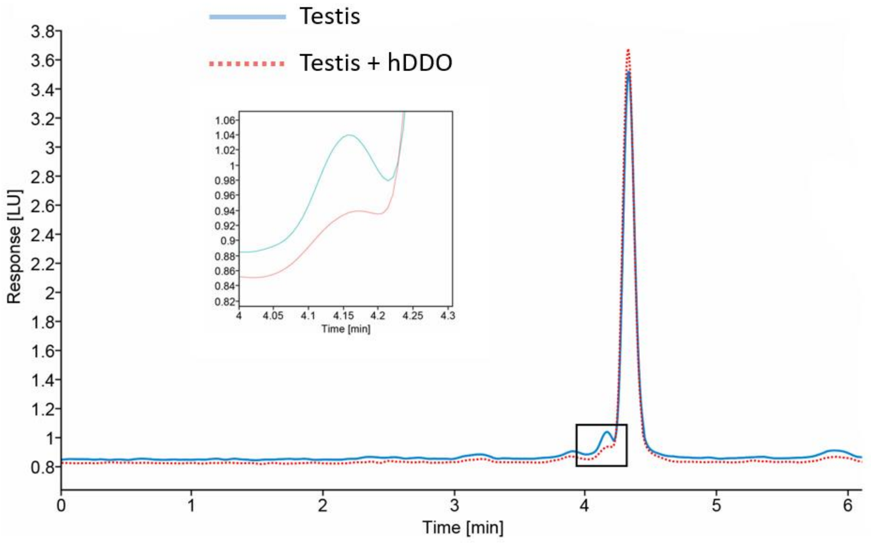

Figure S1. *Representative HPLC chromatogram showing the specificity of D-aspartate peak in the mouse testis.* Overlaid HPLC chromatograms illustrating D-aspartate (D-Asp) and L-Asp peaks obtained from a *R26*$^{+/+}$ mouse testis sample. The identity of the peak corresponding to D-Asp was verified by treating the testis sample with hDDO (inset, red line).
